# Staged neurosurgical approach for giant and progressive neonatal arachnoid cysts: a case series and review of the literature

**DOI:** 10.1007/s00381-024-06385-w

**Published:** 2024-04-11

**Authors:** Aurelia Peraud, Marie Schuler-Ortoli, Matthias Schaal, Frank Reister, Harald Ehrhardt, Ulrike Friebe-Hoffmann

**Affiliations:** 1 Division of Pediatric Neurosurgery, Department of Neurosurgery, University Hospital Ulm, Albert-Einstein-Allee 23, 89081 Ulm, Germany; 2 Section Obstetrics & Perinatology, Department of Obstetrics & Gynecology, University Hospital Ulm, Ulm, Germany; 3 Division of Neonatology and Pediatric Intensive Care Medicine, Department of Pediatrics and Adolescent Medicine, University Hospital Ulm, Ulm, Germany; 4 Department of Radiology, University Hospital Ulm, Ulm, Germany

**Keywords:** Arachnoid cysts, Prenatally diagnosed complex arachnoid cysts, MRI, Neurosurgery

## Abstract

**Objectives:**

Prenatally diagnosed complex arachnoid cysts are very rare. While the true prenatal incidence is still unknown, they account for approximately 1% of intracranial masses in newborns. They rarely exhibit rapid growth or cause obstructive hydrocephalus, but if they increase to such a dimension during pregnancy, the ideal management is not well established. We present our detailed perinatal experience, covering prenatal diagnosis, a compassionate delivery process, and neonatal stabilization. Finally, a thorough postnatal neurosurgical intervention was performed. Initially, our focus was on the gradual reduction of cyst size as a primary effort, followed by subsequent definitive surgical treatment.

**Methods:**

This case series shows the treatment course of three fetuses with antenatally diagnosed large arachnoid cysts. We present pre- and postnatal management and imaging, as well as the surgical treatment plan and the available clinical course during follow-up.

**Results:**

Two girls and one boy were included in the current review. All three cases presented with prenatally diagnosed complex arachnoid cysts that increased in size during pregnancy. The mean gestational age at delivery was 35 weeks (range 32 to 37 weeks), and all patients were delivered by a caesarian section. Increasing head circumference and compression of brain structures were indications for delivery, as they are associated with a high risk of excess intracranial pressures and CSF diapedesis, as well as traumatic delivery and maternal complications. All cysts were supratentorial in location; one expanded into the posterior fossa, and one was a multicompartment cyst. All children underwent an initial surgical procedure within the first days of life. To relieve cyst pressure and achieve a reduction in head circumference, an ultrasound-guided or endoscopic-assisted internal shunt with drainage of the cyst to the ventricles or subdural/subarachnoid space was inserted. Definite surgical therapy consisted of cyst marsupialization and/or cysto-peritoneal shunt implantation. All children survived without severe neurodevelopmental impairments.

**Conclusion:**

With the cases presented, we demonstrate that the slow reduction of immense cyst size as an initial procedure until optimal requirements for final surgical treatment were achieved has proven to be optimal for neurological outcome. Special emphasis has to be taken on the delicate nature of premature newborn babies, and surgical steps have to be thoroughly considered within the interdisciplinary team.

## Introduction

Arachnoid cysts in newborns are very rare and account for 1% of all intracranial lesions in this age group [1, 2]. These cysts represent the majority of intracranial cystic lesions, followed by posttraumatic, porencephalic, or neoplastic cysts. Arachnoid cysts are typically filled with cerebrospinal fluid (CSF)–like fluid and develop within a duplication of the arachnoid layer [3]. They can occur throughout the entire craniospinal axis. Typical locations are at the main fissures (Sylvian, Rolandic, and interhemispheric), at the sella turcica, or in the posterior fossa [4–6]. In most cases, arachnoid cysts develop as single lesions, but they can be associated with other malformations such as dys- or agenesis of the corpus callosum and ventriculomegaly, as well as extracranial abnormalities in the context of a genetic disease [5, 7–9]. Prenatal diagnosis is mainly made after 20 weeks of gestation. While they are more often seen in males, they are mainly located on the left side of the brain. If detected prenatally, these cysts should be followed up by ultrasound or prenatal magnetic resonance imaging (MRI). Fortunately, most cysts are stable in size or regress during pregnancy. However, in around 20% of cases, cysts do enlarge during pregnancy [10].

The number of reported cases in the literature includes less than 50 patients. Our case series of three patients could be a valuable addition to the existing literature. We could demonstrate that stepwise reduction of giant cysts as an initial approach until optimal conditions for definitive surgical treatment are achieved has been shown to be optimal for neurodevelopmental outcome. In addition, we address the special situation of prematurely delivered infants and their associated particular clinical problems (large intracranial cyst accompanied by an oversized head, prematurity-related diseases and associated risks for anesthesia and surgery, fluid balance, temperature control). A staged surgical approach with an acute intervention for gentle reduction of the cyst and head and a final long-term solution is highly recommended. So far, the outcome seems to be not so dismal, with a normal outcome in about 75% of cases [2].

## Methods

We present a series of consecutive cases with an antenatal diagnosis of giant intracranial cysts in three fetuses that were treated at our institution between 2018 and 2023. In all cases, suspected diagnosis was made externally using prenatal ultrasound, and was confirmed, and details were specified by fetal MRI. Additionally, MRI served to confirm diagnosis and assess malformation and brain development in more detail. Typical MRI sequences were T2-weighted images accompanied by T1-weighted images and diffusion-weighted images in some cases. Sonographic reevaluation and monitoring during follow-up were done by 2D/3D ultrasound with a Voluson E10 (GE) device in our institution. Acquired data and longitudinal changes were used to counsel the parents regarding prognosis and treatment options.

The policy at our level 4 perinatal center is to prolong pregnancy as long as possible to avoid complications due to prematurity. This goal must be balanced against the neurological risks caused by the expanding cyst and rise in intracranial pressure. Fortunately, all fetuses showed persistently good intrauterine vital signs and normal fetal movements. Only the calculated head size was crucial. If a fetal head exceeds 40 cm in circumference, delivery even via caesarian section is considered difficult and risky. Therefore, the timing of delivery was determined by the dynamic of the cyst evolution and the intrauterine status of the child.

All children were therefore delivered by a planned caesarean section, and the neonatal team first stabilized the newborn after birth.

On the first or second day of life, the neonates had a thorough neurological examination by a senior pediatric neurologist and, additionally, a brain MRI (3 T, Siemens Erlangen) under spontaneous sleep (“feed and wrap”) in a vacuum mattress. Standard sequences were T2-weighted turbo spin echo (TSE), T1-weighted gradient echo (FLASH = fast low-angle shot), susceptibility-weighted imaging (SWI), and diffusion tensor imaging (DTI). If possible, high-resolution T2-weighted sequences with isometric voxels (CISS = constructive interference in steady state) were added. The MRI served as a basis for further clinical and surgical decision-making. Follow-up MRIs were done either as described above or alternatively with fast T2-weighted sequences (HASTE = half-Fourier acquisition single-shot turbo spin echo imaging) in spontaneous sleep without sedation.

## Results/Case series

### Case 1

A 41-year-old fourth gravida first para presented to our center at 31 + 1 weeks of gestation p.m. (post-menstrual) with a fetus who had a large suprasellar arachnoid cyst.

**Fig. 1:**
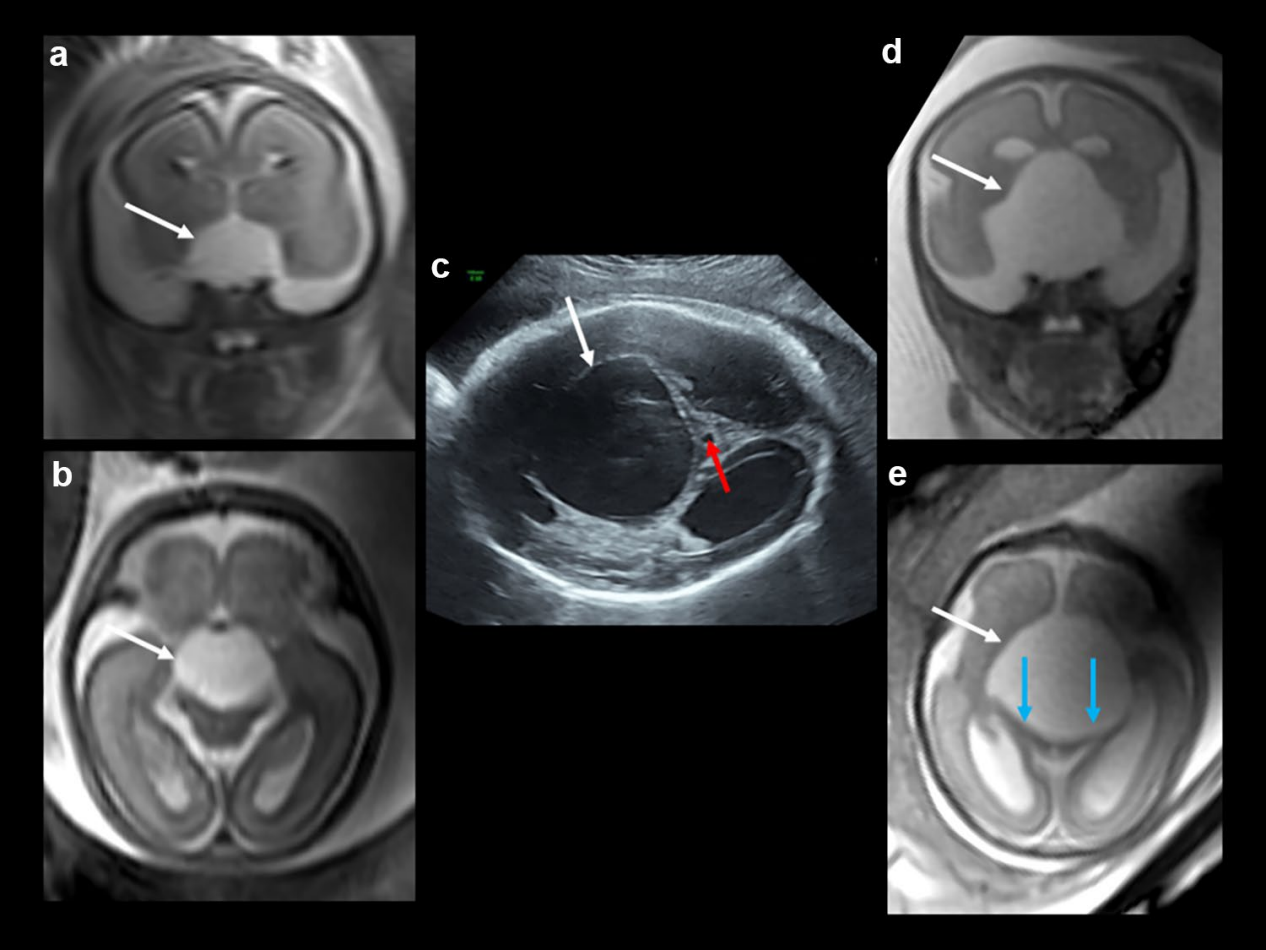
**a**, **b** Prenatal MRI at 21 weeks of gestational age (T2-weighted) in coronal (**a**) and in axial (**b**) orientations (courtesy of PD Dr. K. Kreiser, Technical University, Munich): suprasellar midline cystic formation (white arrows) with moderate displacing character. **c** Prenatal ultrasound at 31 + 1 weeks of gestational age: suprasellar arachnoid cyst (white arrow) of up to 5-cm diameter with displacement of the surrounding brain structures and occlusion of the aqueduct (red arrow) with subsequent hydrocephalus. **d**, **e** Prenatal MRI at 26 weeks of gestational age (T2-weighted) in coronal (**d**) and in axial (**e**) orientations (courtesy of PD Dr. K. Kreiser, Technical University, Munich): significant increase in size of the suprasellar cystic formation (white arrows) now with clear displacement of the cerebral peduncles (**e**, blue arrows)

After a first trimester screening with an adjusted risk for trisomy 21 of 1:71 without any further invasive diagnostics, the patient underwent a specialized organ screening at 22 weeks. A cystic formation was described in the posterior fossa. Subsequent amniocentesis prevailed a normal karyotype. Repeated sonographic evaluations at different specialized centers in Germany and three fetal MRIs demonstrated a large fetal suprasellar arachnoid cyst growing over time to 5 cm with displacement of surrounding brain structures, including the brainstem and corpus callosum, as well as occlusion of the aqueduct with consecutive hydrocephalus (Fig. 1). The parents opted against termination of the pregnancy, and they finally presented at our center with the explicit wish for active care and postnatal neurosurgical treatment. As advised by an interdisciplinary board of prenatal diagnosticians, obstetricians, neonatologists, and pediatric neurosurgeons, the pregnancy was closely monitored.

Primary caesarian section was performed at 33 + 4 weeks of gestation due to an increasing head circumference, the onset of brain sparing, and an increase in the Vmax of the middle cerebral artery. The preterm infant was born with a weight (W) of 2685 g, a body length (L) of 44.0 cm, and a head circumference (HC) of 33.5 cm (89th percentile). APGAR scores were 8/9/9 at 1, 5, and 10 min of life, and umbilical cord artery pH was 7.39. Postpartum, the infant was immediately taken over by the neonatology team and admitted to the NICU.

**Fig. 2:**
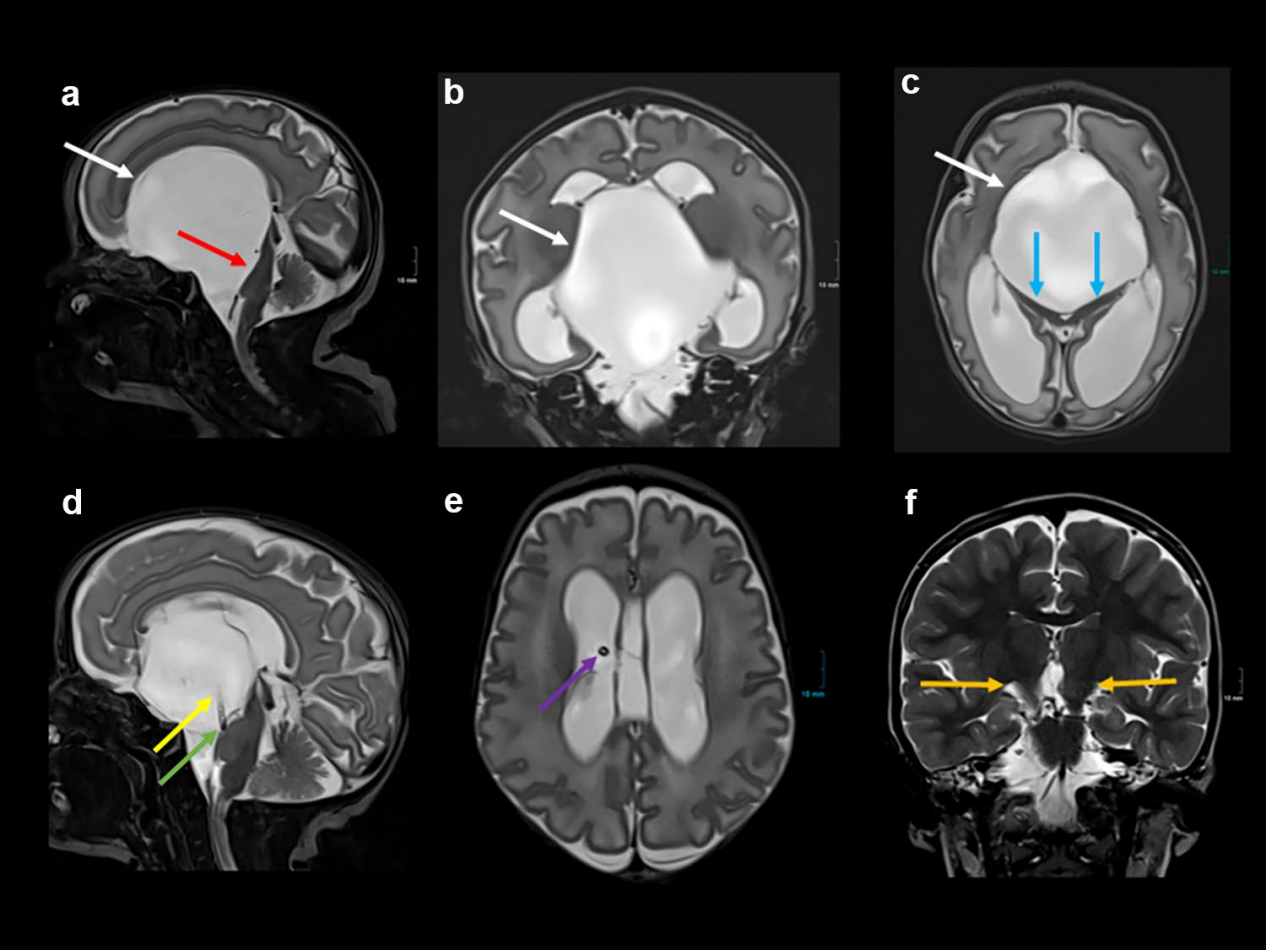
**a**–**c** Postnatal MRI (T2 TSE) in sagittal (**a**), coronal (**b**), and axial (**c**) orientations: large midline cystic formation (white arrows) with compression of the brainstem (**a**, red arrow) and the cerebral peduncles (**c**, blue arrows) causing significant hydrocephalus. **d**, **e** MRI (T2 TSE) in sagittal (**d**) and axial (**e**) orientations 1 month after endoscopic fenestration of the cyst. The implanted internal shunt catheter connects the cyst (**d**, yellow arrow) with the prepontine cistern (**d**, green arrow) and the lateral ventricle (**e**, purple arrow). Consecutive reduction of cyst size and hydrocephalus. **f** MRI at the age of 4 years (T2 TSE in coronal orientation; with friendly approval of Dr. Hundt, Radiology Munich Nymphenburg): subtotal regression of the cyst and no hydrocephalus with VP-Shunt (not shown), some asymmetry of the cerebral peduncles (orange arrows)

The postnatal MRI showed a large midline cyst compressing the cerebral peduncles and causing significant hydrocephalus (Fig. 2). The child was stable in vital signs and displayed no neurological symptoms, and neurosurgery was scheduled for the sixth day of life. After endoscopic fenestration of the cyst, an internal shunt catheter with additional holes was inserted, connecting the cyst with the prepontine cistern and the lateral ventricle. One month after surgery, a reduction in cyst size and hydrocephalus was evident on follow-up MRI with repeated punctures of the connected subcutaneous Ommaya reservoir (Fig. 2). Unfortunately, the cyst fenestration was not sufficient, and the catheter slipped out of the prepontine cistern after 4 months with a new and sudden percentile-crossing increase of the head circumference. A connection of the existing catheter to a VP shunt system was made during emergency intervention. A programmable gravitational valve was implanted. Another 4 months after the latest intervention, and at 4 years of age, MRI confirmed sufficient cystic drainage with an almost complete disappearance of the cyst (Fig. 2). The psychomotor development of the girl at the age of 4 is completely normal, and she reached all milestones on time.

### Case 2

During second trimester screening, a 10-mm cystic structure was seen in the right posterior fossa of the fetus of a 39-year-old third gravida second para. Several sonographic follow-ups and two fetal MRIs (outside our institution) confirmed the diagnosis of a large arachnoid cyst mainly in the right posterior fossa with supratentorial extension. A marked increase in size over time was documented, with displacement of the cerebellum, mesencephalon, and compression of the aqueduct with consecutive development of a massive hydrocephalus. On MRI, gyration was judged to be rather reduced.

**Fig. 3:**
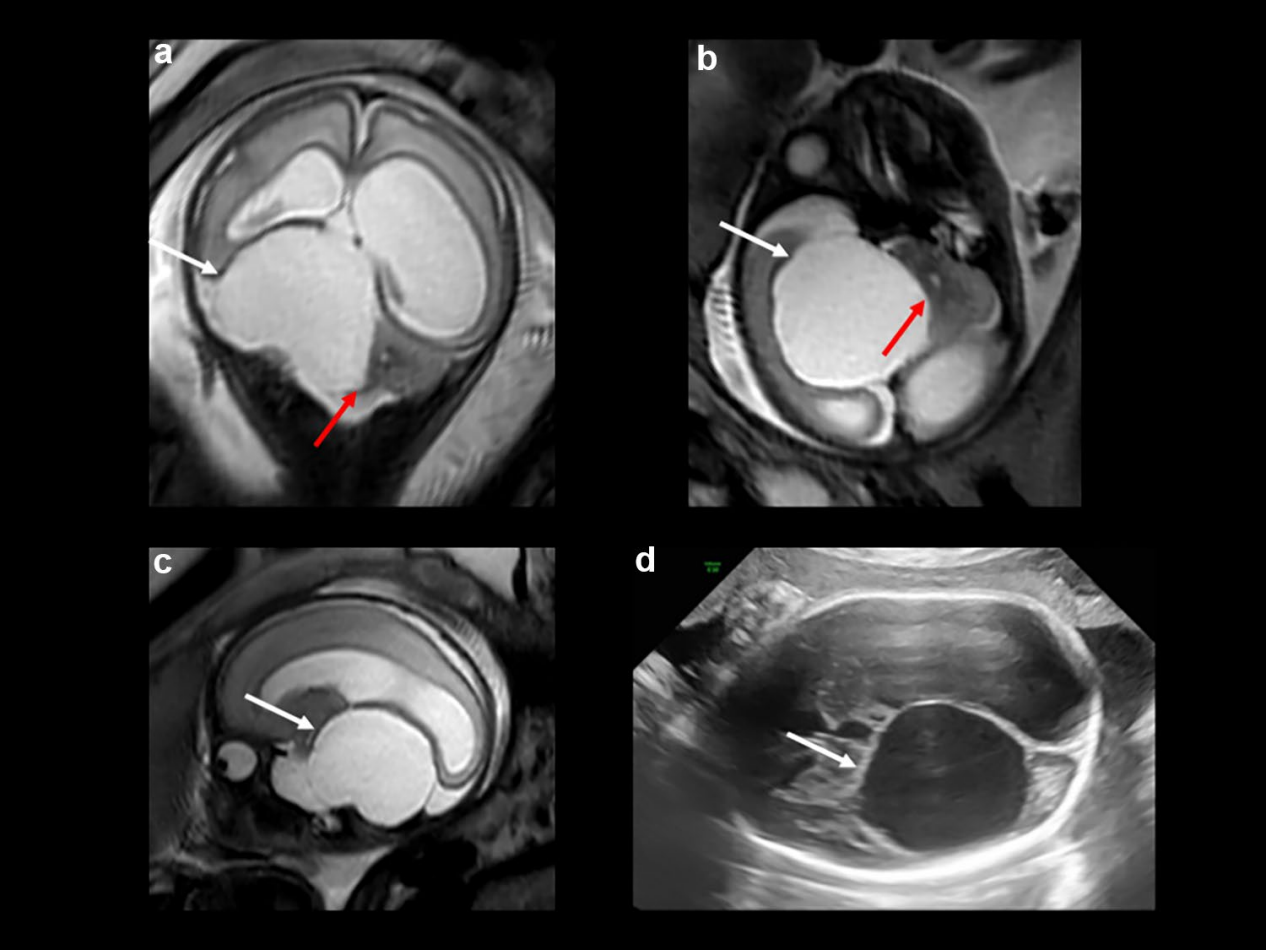
**a**, **b**, **c** Prenatal MRI at 27 weeks of gestational age (T2-weighted) in coronal (**a**), axial (**b**), and sagittal (**c**) orientations (courtesy of PD Dr. D. M. Hedderich, PD Dr. T. Finck, and Prof. Dr. J. S. Krischke, Technical University, Munich): large right-sided cystic formation with supratentorial and infratentorial extension (white arrows) with significant displacement of the right cerebellar hemisphere (**a**, **b** red arrows) and supratentorial hydrocephalus. **d** Prenatal ultrasound at 30 + 3 weeks of gestational age showing a large arachnoid cyst (white arrow) in the right posterior fossa with supratentorial extension and displacement of the cerebellum, mesencephalon, and compression of the aqueduct with consecutive development of a pronounced hydrocephalus

**Fig. 4:**
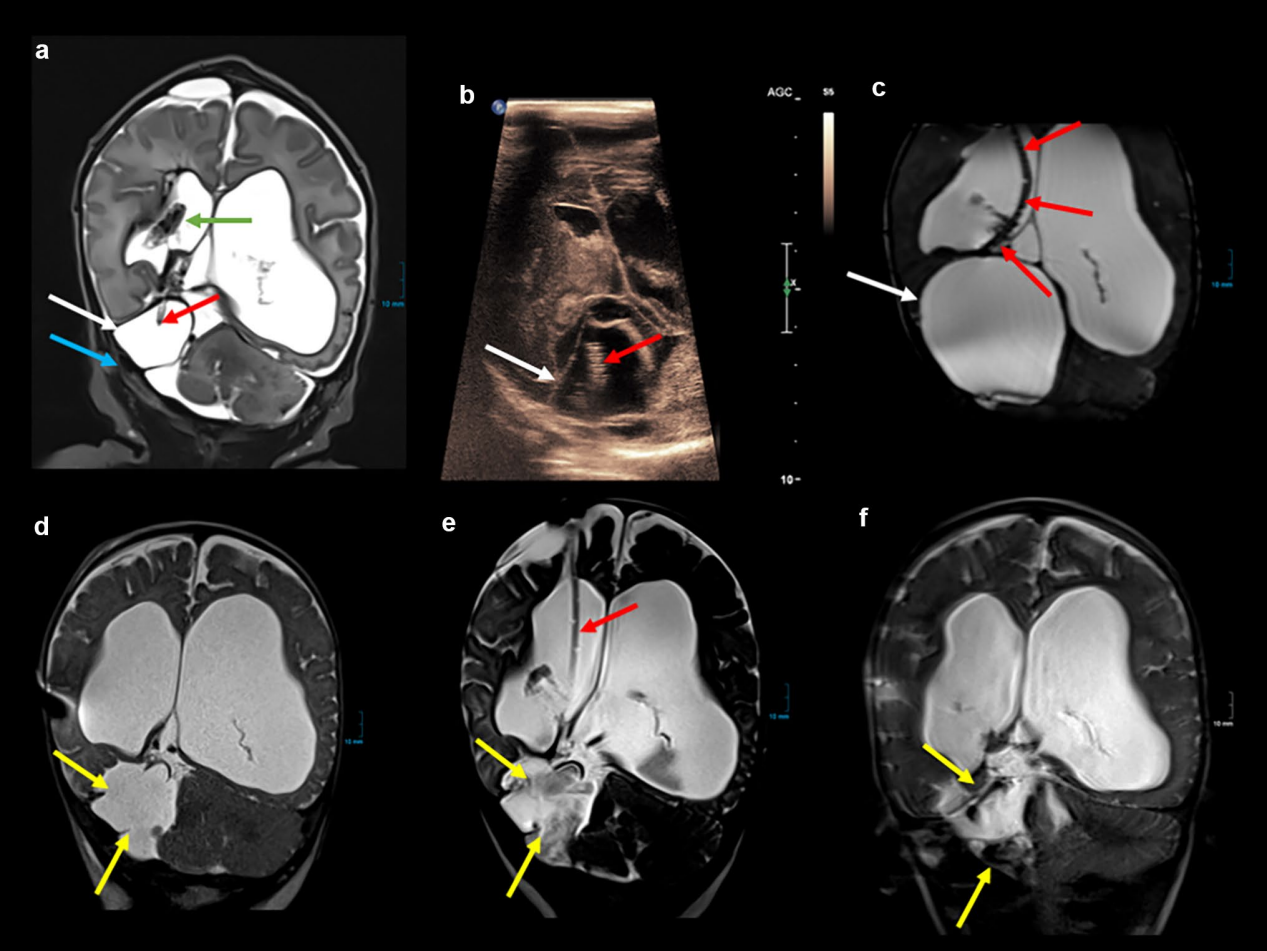
**a**, **b** MRI (**a**: T2 TSE) and transfontanellar ultrasound (**b**) both in coronal orientations after surgery: ventricular catheter (red arrow) with connection of the cyst (white arrow) with the right lateral ventricle with some bleeding into the ventricle (green arrow); the cyst has developed at the level of the tentorium next to the transverse sinus (blue arrow). **c** MRI (T2 CISS in coronal multiplanar reconstruction) at 3 months with recurrence of the cyst (white arrow) because the catheter slipped out of the cyst (red arrows). **d** MRI (T2 HASTE in coronal orientation) after supratentorial minicraniotomy with fenestration of the cyst (supra- and infratentorial, yellow arrows), consecutive reduction in cyst size. **e** MRI (T2 HASTE in coronal orientation): due to persistent enlargement of the supratentorial ventricular system, a VP shunt with connection to the existing ventricular catheter (red arrow) was established. The fenestration of the cyst (supra- and infratentorial, yellow arrows) is still evident. **f** Follow-up MRI (T2 HASTE in coronal orientation) at the age of 18 months, with e-vacuo enlargement of the lateral ventricles, the fenestration of the cyst with consecutive flow void artifacts of cerebrospinal fluid (yellow arrows) is still evident

At 30 + 3 weeks of gestation, fetal ultrasound examination revealed that the cyst had grown up to 6.4 cm, the lateral ventricles dilated to 22 mm on the right and 47 mm on the left side, and the cerebellum was markedly displaced to the left (Fig. 3). Due to suspected brainstem compression with abruptly reduced intrauterine movements of the child, the decision for delivery was made in the interdisciplinary board. After a completed course of antenatal steroids, a primary caesarean section was performed at 32 + 5 weeks of gestation. The birth measurements of the preterm male infant were W 2410 g, L 46 cm, HC 36.5 cm (> 99th percentile), APGAR scores 3/8/10, and umbilical cord artery pH 7.36. This child was also cared for immediately after delivery by the neonatal intensive care team. The respiratory and cardiovascular adaptation of the infant in the delivery room was stable, and the infant was transferred to the NICU on non-invasive respiratory support. Due to the prenatal findings and significant brainstem compression, verified by head ultrasound, and respiratory problems, we opted for surgery without a preoperative MRI on the day of birth. Under intraoperative sonographic guidance, a ventricular catheter with additional holes was introduced via a frontal/transfontanellar route to connect the cyst with the right lateral ventricle. During surgery, it was noted that the cyst wall was rather firm and difficult to perforate. Obviously, there was some hemorrhage into the ventricle detected on postoperative head ultrasound and MRI of the brain, but IVH resorbed without residuals, the cyst continued to decrease in dimension, and the hydrocephalus was relieved. At 3 months of age, the parents observed again a sudden increase in head circumference, and the follow-up MRI showed a recurrence of the cyst because the catheter had slipped out of the cyst. It turned out on this MRI that the cyst had developed within the tentorium, and the transverse sinus was running exactly in between both dural layers (Fig. 4). Therefore, a careful supratentorial mini craniotomy was performed, and the cyst within the tentorium was fenestrated into both compartments (supra- and infratentorial) under microsurgical means. Follow-up MRI images confirmed the effective fenestration of the cyst and a reduction in size, but the boy showed persistent macrocephalus and enlargement of the supratentorial ventricular system. He finally received a VP shunt with connection to the existing ventricular catheter and implantation of a programmable gravitational valve at the age of 6 months (Fig. 4). At 24-month follow-up, his neurocognitive and motor development was unremarkable.

### Case 3

**Fig. 5:**
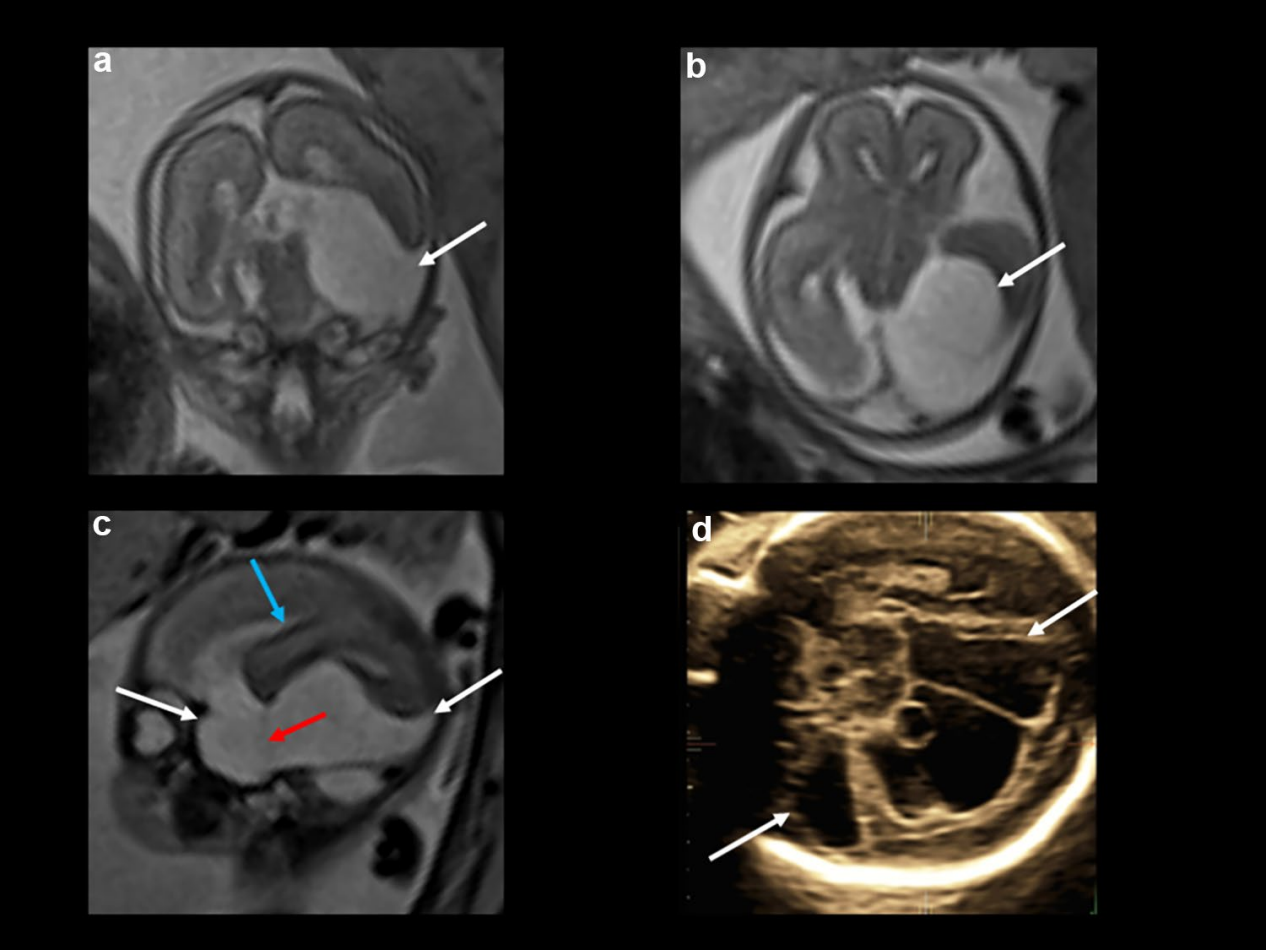
**a**, **b**, **c** Prenatal MRI at 22 weeks of gestational age (T2-weighted) in coronal (**a**), axial (**b**), and sagittal (**c**) orientation (courtesy of Prof. Dr. S. Stöcklein, Ludwig-Maximilians-University, Munich): left-sided supratentorial cystic formation (white arrows) with recognizable septations (**c**, red arrow) and moderate displacement of the surrounding structures, especially the left temporal lobe (**c**, blue arrow). **d** Prenatal ultrasound at 25 + 6 weeks of gestational age: large 4-chambered arachnoid cyst (white arrows) of 7 cm in diameter without any signs of hydrocephalus or brainstem compression (approval of Dr. K. Lato, University Hospital Ulm)

A 30-year-old second gravida first para was pregnant with a female fetus, in whom sonographically a 3-chambered, 4-cm large cystic lesion was externally detected by ultrasound in the parieto-temporo-occipital region of the left hemisphere, and the adjacent temporal tissue was absent. The suspicion of a large chambered arachnoid cyst was confirmed by a fetal MRI (done at another institution). The mother was sent to our obstetric department for further counseling. In the meantime, the arachnoid cyst had grown to 7 cm in diameter without any signs of hydrocephalus or brainstem compression (Fig. 5). The patient was delivered by elective caesarean section at 37 + 4 weeks of gestation with a fetal head size > 97th percentile. Perinatal data were as follows: W 3295 g, L 51 cm, HC 38 cm (> 99th percentile), APGAR scores 9/10/10, and umbilical cord artery pH 7.36.

**Fig. 6:**
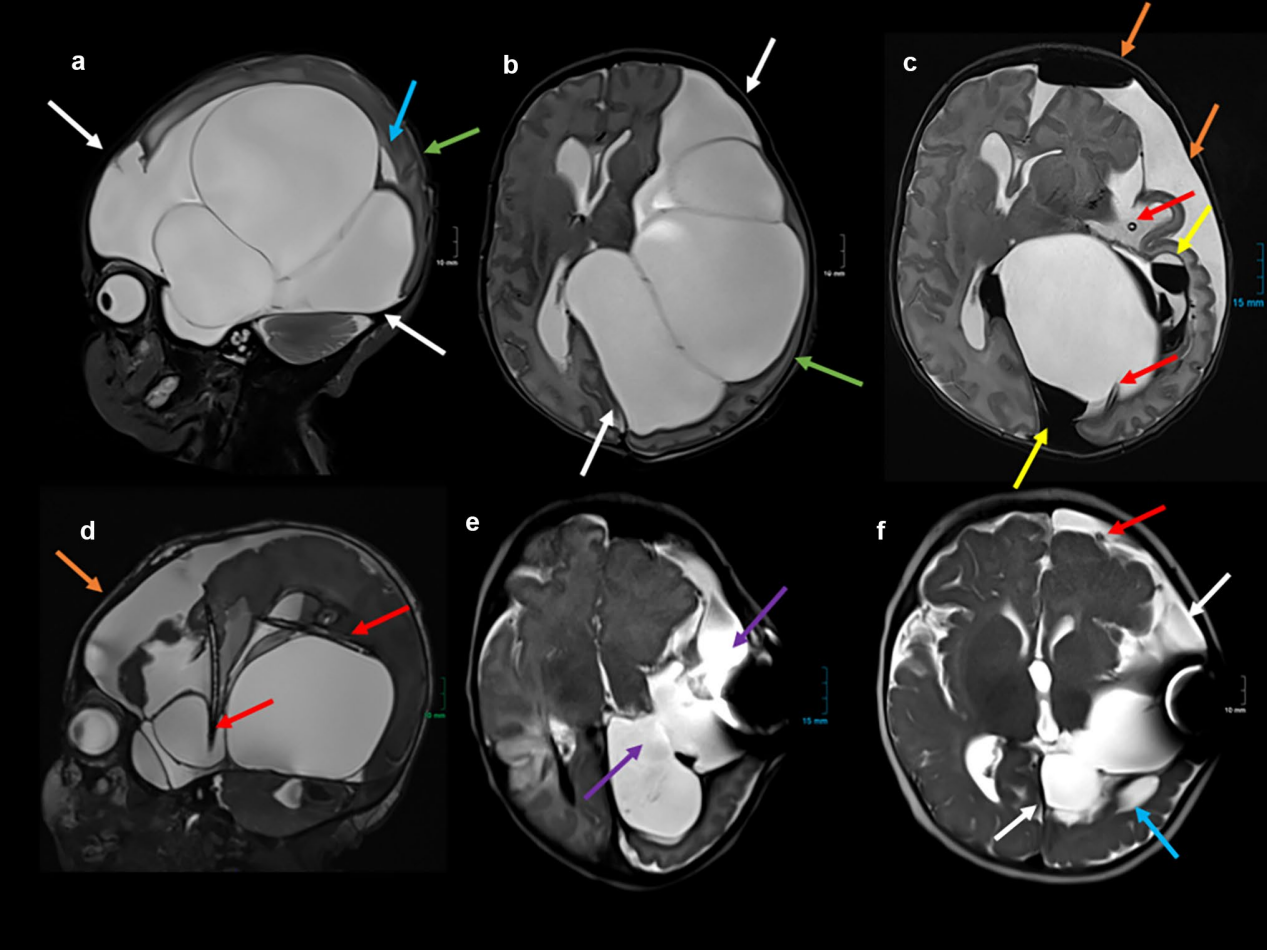
**a**, **b** Pre-surgical MRI (T2 TSE in sagittal (**a**) and axial (**b**) orientations): several large cysts occupying most of the left hemispheric space (white arrows). Dislocation of the left temporal lobe (**a**, **b**, green arrow) and left temporal horn (**a**, blue arrow). **c**, **d** MRI after surgery (**c**: T2 TSE in axial orientation, **d**: T2 CISS in sagittal multiplanar reconstruction): two ventricular catheters in several compartments of the cystic formation (red arrows) with some amount of hemorrhage (yellow arrows), left frontal hygroma (orange arrows) with pneumatocephalon. **e** MRI (T2 HASTE in axial orientation) after microsurgical marsupialization of the cyst walls with consecutive communication of the compartments with each other (purple arrows). **f** Follow-up MRI (T2 HASTE in axial orientation) at the age of 7 months: some residuals of the cysts (white arrows), left temporal horn still dislocated (blue arrow), residual left frontal hygroma with subduro-peritoneal shunt (red arrow)

Despite the immense head circumference, the baby girl adapted well postnatally. On preoperative MRI scans, several large cysts became visible, occupying most of the hemispheric space on the left side (Fig. 6). On the second day of life, a sonographic and endoscopic-assisted implantation of two ventricular catheters (with additional holes to reach all cysts) connected to one Rickham reservoir was performed, intended to drain all four cysts. During the subsequent days, with almost daily reservoir punctures, the cysts gradually became smaller, and the distorted anatomy became more obvious. Finally, almost 4 weeks after the first intervention, and with a body weight of more than 3 kg, the female infant underwent microsurgical cyst marsupialization through a left frontotemporal mini-craniotomy and the implantation of a subduro-peritoneal shunt with a programmable gravitational valve. During the first 10 months, the girl showed normal psychomotor development but developed focal seizures and required antiepileptic therapy.

## Discussion

There are only a few reports in the literature on the management of neonatal or even prenatally diagnosed arachnoid cysts (Table 1). In most cases, these cysts are diagnosed during second trimester ultrasound screening (62%) [11, 12]. But in the series by De Keersmaecker et al. 75% were detected during the third trimester screening. Cysts of the posterior fossa were detected earlier than supratentorial cysts because they cause early CSF pathway obstruction, leading to hydrocephalus [11, 13].

**Table 1:** Published reports of prenatally detected arachnoid cysts

**Reference**	**Year**	**Trimester at diagnosis**	**Cyst location**	**No of pts.**	**Age at surgery**	**Type of surgical intervention**	**Revision surgery**
Fuchs et al. [26]	2008	3rd	interhemispheric	1	5 w	Cystoperitoneal shunt	Shunt revision at 8 m
Haino et al. [22]	2009	3rd	quadrigeminal plate	1	2 w	Cyst puncture and cystoperitoneal shunt	-
Gedikbasi et al. [24]	2010	2nd	suprasellar	1	2 w	Endoscopic fenestration	Infection Redo endoscopic fenestration
Chalouhi et al. [16]	2012	2nd	suprasellar	1	31 gestational week	Fetoscopic cystocisternostomy	Endoscopic fenestration postnatal
De Keersmaecker et al. [11]	2015	4 at 2nd 8 at 3rd	9 supratentorial 3 infratentorial	12	1 at 4 d 1 at 7 d 1 at 2 m 1 abortion	Endoscopic fenestration 2 (cysts with max. diameter of 37 and 53 mm) Cyst drainage 1 Conservative treatment 8	-
Yahal et al. [2]	2019	3rd	9 supratentorial 20 infratentorial	29	n.a	VP shunt 2 Microsurgical fenestration 1 Spontaneous resolution 9	n.a
Soleman et al. [20]	2021	n.a	23 infratentorial	23 (35)*	At a mean of 6 m	Microsurgical fenestration 10 Endoscopic fenestration 7 VP shunt 2 Combined surgeries 8**	30%
Endo et al. [23]	2022	2nd	prepontine	1	3 m	Microsurgical fenestration	n.a

*n.a., *not available

^*^The whole cohort of 35 patients included pre- and postnatally diagnosed arachnoid cysts

^**^Combined surgeries included open microsurgical fenestration and ETV in 1, endoscopic fenestration and ETV in 5, and endoscopic fenestration and shunt in 2 patients, respectively

Fortunately, most cysts stabilize during fetal development or even show regression [14]. In the series by Grossman et al. more than 80% of prenatally diagnosed arachnoid cysts were no longer visible on follow-up images, but those who progressed were larger than 2 cm at the initial diagnosis [12]. According to Youssef et al. fetal cysts do grow in 20 to 23.9% [10]. To our knowledge, there exists no other report on extremely large and complex arachnoid cysts in newborn infants that required rapid intervention. Most children were treated within the first months of life [11] (Table 1).

Not uncommon are additional anomalies such as ventriculomegaly, corpus callosum agenesis, heterotopia, and aqueduct stenosis associated with arachnoid cysts in this age group (in up to 74% of the cases) [10]. Due to the immense size of the cysts in our case series, the normal anatomy was completely distorted and could only be reconstructed after cyst decompression.

The critical issue during the prenatal visits was defining the ideal timing of delivery. As mentioned before, delivery should be as late as possible to reduce the risks of prematurity, but this has to be weighed against the risk of intrauterine compromise of cerebral structures due to the expanding cyst. Frequent assessments via ultrasound scanning were necessary to document cyst enlargement and define the perfect timing of delivery*.* A planned cesarean section offers several advantages over attempting vaginal delivery. The enlarged fetal head is likely to result in obstructed labor. This may increase the risk of additional traumatic injuries to the head and the brain. Moreover, scheduled cesarean sections will facilitate the presence of skilled and trained personnel at the time of delivery [15].

Our series of patients comprises complex giant arachnoid cysts in three different locations, and the decision to deliver and treat them was mainly driven by the dramatic intrauterine cyst evolution. The first child had a progressive suprasellar arachnoid cyst, and André et al. published a new classification system and described three different types (SAC-1 to SAC-3) depending on the direction of cyst extension (upward, downward, or lateral/asymmetrical). In their series of 35 patients, 14 were diagnosed prenatally, but none of them was delivered before term, and one child was sent for fetoscopic surgery (the case presented by Chalouhi et al. [16, 17]). Other classifications exist for middle cranial fossa cysts according to Galassi (types I to III) [18]. Cysts of the posterior fossa are very rare (4.6%) [19] and are classified according to their location (quadrigeminal, retrocerebellar, supracerebellar, cerebello-pontine angle, prepontine) [20–23].

Known differential diagnoses of neonatal arachnoid cysts are porencephalic cysts, choroid plexus cysts, glioependymal cysts, status post hemorrhage, and in the posterior fossa Blake‘s pouch cyst, Dandy Walker malformation, and megacisterna magna. Arachnoid cysts that are associated with other congenital abnormalities should be screened for genetic syndromes (e.g., Aicardi syndrome or Chudley-McCullough syndrome); isolated cysts are rarely associated with a genetic disorder [11, 24].

**Fig. 7:**
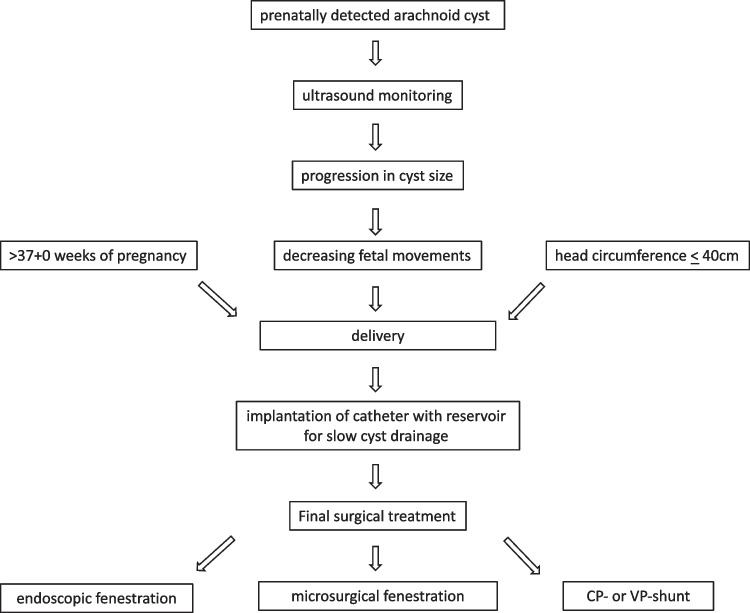
Treatment algorithm

In our current series of three antenatally diagnosed cysts, all showed progressive enlargement during intrauterine development and required rapid intervention after delivery. The initial surgical approach was as minimally invasive as possible to obtain quick release of the pressure due to the cyst size and to ensure normal adaptation of the newborn child. The endoscopically or sonographically guided insertion of a ventricular catheter was intended to create internal cyst drainage into the ventricle or the (prepontine) cistern. However, all cysts required sooner or later repeated punctures of the attached subcutaneous Rickham reservoir due to persistent head size and/or distension of the fontanelle or sutures. Punctures were executed between three times per week and up to daily punctures with a drain volume of 10 ml/kg body weight for 2 to 4 weeks. In none of the infants, a nosocomial infection occurred. Final cyst drainage was accomplished during the second surgical intervention at a later stage when the infant was well-adapted and neurologically stable. Our treatment algorithm is given in Fig. 7.

Several surgical procedures have been described in the literature to deal with arachnoid cysts, including decompressive craniectomy, cyst marsupialization, cyst fenestration, endoscopic cystocisternostomy, or cystoventriculostomy, and cystoperitoneal shunts [17, 25] (Table 1).

A detailed analysis of the surgical procedures, results, and complications was given by Fuchs et al. and Spennato et al. for interhemispheric or quadrigeminal cysts [25, 26]. In the review of Fuchs, 27 children out of 44 with interhemispheric cysts underwent cystoperitoneal shunting. Five years after the initial surgery, 74% of the children showed normal neurological development, 22% had psychomotor delays, and one child had died [26]. Shunt revision was necessary in 41% of these cases. Cyst resection was performed in 17 out of 44 children. In 87% of infants, one surgical procedure was sufficient and yielded a comparable good outcome as with the shunt procedure [26]. Spennato et al. favored endoscopic cyst fenestration with equal success rates for children at any age, but the review does not focus on prenatally diagnosed giant arachnoid cysts, which require immediate treatment after delivery. A single case report from Chalouhi et al. showed the successful fetoscopic fenestration/feto-cisternoscopy of a progressive suprasellar cyst at the 31st week of gestation. The baby was born at 36 weeks due to premature rupture of the membranes and required an additional surgical intervention for definite cyst drainage after birth [16]. A summary of the existing literature on prenatally diagnosed arachnoid cysts and surgical treatments is given in Table 1. Only in the series by De Keersmaecker et al. is an early intervention in two cases at 1 week reported.

An initial cystoperitoneal shunt in our patients with their extremely large heads was considered to carry the risk of cyst overdrainage and the development of subdural hygromas or hematomas despite the use of programmable gravitational valves. In addition, the skin of the head was too thin to tolerate the implantation of extensive foreign material. Therefore, the gradual reduction of the head size, a more solid skin situation, and a child weighing over 3 kg were achieved with the precise implantation of catheters into the cysts, which were connected to a subcutaneous reservoir for subsequent sterile punctures (Fig. 7). Even though the three children required additional surgical procedures for final cyst treatment, their neurocognitive and motor developments were good to excellent. Repeated surgical interventions are not uncommon in reported cases (Table 1) and rise to 30% for infratentorial cysts, as shown in the series of Soleman [20].

Although the present case series has limitations due to the low number of cases and the retrospective nature, we found our experience and our surgical approach for these progressive and large arachnoid cysts in neonates with the pediatric neurosurgical community worth sharing.

## Conclusion

With the experience of the presented case series, we pursue the policy of a staged surgical procedure. The first surgery, which is usually done within hours to days after delivery, should be minimally invasive to reduce the volume of the cyst and correct the ventricular enlargement. With the repetitive punctures through the reservoir, the oversized head with bulging fontanelles and distended sutures can gradually be downsized. Finally, with the decompression of the cyst, the formerly distorted anatomy becomes clearer, and a definite surgical intervention can be planned accordingly.

## Data Availability

No datasets were generated or analyzed during the current study.

## References

[1] Pappalardo EM, Militello M, Rapisarda G, Imbruglia L, Recupero S, Ermito S (2009). Fetal intracranial cysts: prenatal diagnosis and outcome. J Prenat Med.

[2] Yahal O, Katorza E, Zvi E, Berkenstadt M, Hoffman C, Achiron R (2019). Prenatal diagnosis of arachnoid cysts: MRI features and neurodevelopmental outcome. Eur J Radiol.

[3] Westermaier T, Schweitzer T, Ernestus RI (2012). Arachnoid cysts. Adv Exp Med Biol.

[4] Al-Holou WN, Yew AY, Boomsaad ZE, Garton HJ, Muraszko KM, Maher CO (2010). Prevalence and natural history of arachnoid cysts in children. J Neurosurg Pediatr.

[5] Jünger ST, Knerlich-Lukoschus F, Röhrig A, Al Hourani J, Kunze S, Eberle J (2022). Clinical variety and prognosis of intracranial arachnoid cysts in children. Neurosurg Rev.

[6] Oberbauer RW, Haase J, Pucher R (1992). Arachnoid cysts in children: a European co-operative study. Childs Nerv Syst.

[7] Carbone J, Sadasivan AP (2021). Intracranial arachnoid cysts: review of natural history and proposed treatment algorithm. Surg Neurol Int.

[8] McLaurin-Jiang SV, Wood JK, Crudo DF (2016). Septooptic dysplasia with an associated arachnoid cyst. Case Rep Pediatr.

[9] Silva Baticam N, Aloy E, Rolland A, Fuchs F, Roujeau T (2022). Prenatally symptomatic suprasellar arachnoid cyst: when to treat? A case-base update. Neurochirurgie.

[10] Youssef A, D'Antonio F, Khalil A, Papageorghiou AT, Ciardulli A, Lanzone A (2016). Outcome of fetuses with supratentorial extra-axial intracranial cysts: a systematic review. Fetal Diagn Ther.

[11] De Keersmaecker B, Ramaekers P, Claus F, Witters I, Ortibus E, Naulaers G (2015). Outcome of 12 antenatally diagnosed fetal arachnoid cysts: case series and review of the literature. Eur J Paediatr Neurol.

[12] Grossman TB, Uribe-Cardenas R, Radwanski RE, Souweidane MM, Hoffman CE (2022). Arachnoid cysts: using prenatal imaging and need for pediatric neurosurgical intervention to better understand their natural history and prognosis. J Matern Fetal Neonatal Med.

[13] Gandolfi Colleoni G, Contro E, Carletti A, Ghi T, Campobasso G, Rembouskos G (2012). Prenatal diagnosis and outcome of fetal posterior fossa fluid collections. Ultrasound Obstet Gynecol.

[14] Pierre-Kahn A, Hanlo P, Sonigo P, Parisot D, McConnell RS (2000). The contribution of prenatal diagnosis to the understanding of malformative intracranial cysts: state of the art. Childs Nerv Syst.

[15] Ruprai CK, Pring DW, Vipond A, Duffey P (2007). Pregnancy complicated by a large intracranial arachnoid cyst: multidisciplinary approach to safe delivery. J Obstet Gynaecol.

[16] Chalouhi GE, Marangoni M, Zerah M, Ville Y (2012) Intrauterine treatment of a suprasellar inter-hemispheric arachnoid cyst by feto-cisternoscopy. Poster Abstract P09.24. 22nd World Congress on Ultrasound in Obstetrics and Gynecology. Ultrasound Obstet Gynecol 171–310

[17] André A, Zérah M, Roujeau T, Brunelle F, Blauwblomme T, Puget S (2016). Suprasellar arachnoid cysts: toward a new simple classification based on prognosis and treatment modality. Neurosurg.

[18] Galassi E, Tognetti F, Gaist G, Fagioli L, Frank F, Frank G (1982). CT scan and metrizamide CT cisternography in arachnoid cysts of the middle cranial fossa: classification and pathophysiological aspects. Surg Neurol.

[19] Takeshige N, Eto T, Nakashima S, Sakata K, Uchikado H, Abe T (2018). Rare case of a rapidly enlarging symptomatic arachnoid cyst of the posterior fossa in an infant: a case report and review of the literature. Surg Neurol Int.

[20] Soleman J, Kozyrev DA, Constantini S, Roth J (2021). Surgical treatment and outcome of posterior fossa arachnoid cysts in infants. J Neurosurg Pediatr.

[21] Erdinçler P, Kaynar MY, Bozkus H, Ciplak N (1999). Posterior fossa arachnoid cysts. Br J Neurosurg.

[22] Haino K, Serikawa T, Kikuchi A, Takakuwa K, Tanaka K (2009). Prenatal diagnosis of fetal arachnoid cyst of the quadrigeminal cistern in ultrasonography and MRI. Prenat Diagn.

[23] Endo M, Usami K, Masaaki N, Ogiwara H (2022). A neonatal purely prepontine arachnoid cyst: a case report and review of the literature. Childs Nerv Syst.

[24] Gedikbasi A, Palabiyik F, Oztarhan A, Yildirim G, Eren C, Ozyurt SS (2010). Prenatal diagnosis of a suprasellar arachnoid cyst with 2- and 3-dimensional sonography and fetal magnetic resonance imaging: difficulties in management and review of the literature. J Ultrasound Med.

[25] Spennato P, Ruggiero C, Aliberti F, Buonocore MC, Trischitta V, Cinalli G (2013). Interhemispheric and quadrigeminal cysts. World Neurosurg.

[26] Fuchs F, Moutard ML, Blin G, Sonigo P, Mandelbrot L (2008). Prenatal and postnatal follow-up of a fetal interhemispheric arachnoid cyst with partial corpus callosum agenesis, asymmetric ventriculomegaly and localized polymicrogyria. Case report Fetal Diagn Ther.

